# N-Terminal pro-B-type natriuretic peptide and coronary microvascular dysfunction in women with preserved ejection fraction: A report from the Women’s Ischemia Syndrome Evaluation–Coronary Vascular Dysfunction (WISE-CVD) study

**DOI:** 10.1371/journal.pone.0243213

**Published:** 2020-12-03

**Authors:** Erika Jones, Janet Wei, Michael D. Nelson, May Bakir, Puja K. Mehta, Chrisandra Shufelt, Margo Minissian, Behzad Sharif, Carl J. Pepine, Eileen Handberg, Galen Cook-Wiens, George Sopko, C. Noel Bairey Merz

**Affiliations:** 1 Barbra Streisand Women’s Heart Center, Cedars-Sinai Heart Institute, Los Angeles, CA, United States of America; 2 University of Florida, Gainesville, FL, United States of America; 3 Samuel Oschin Comprehensive Cancer Institute, Cedars-Sinai Medical Center, Los Angeles, CA, United States of America; 4 National Heart, Lung, and Blood Institute, Bethesda, MD, United States of America; Scuola Superiore Sant'Anna, ITALY

## Abstract

**Background:**

Women with symptoms and signs of ischemia, preserved left ventricular ejection fraction (LVEF), and no obstructive coronary artery disease (CAD), often have coronary microvascular dysfunction (CMD), and are at risk of future heart failure with preserved ejection fraction (HFpEF). N-terminal pro-B-type natriuretic peptide (NT-proBNP) is used to evaluate HF and myocardial ischemia. Relationships between NT-proBNP and CMD are not well defined in this population.

**Methods:**

We evaluated resting NT-proBNP levels in 208 women with symptoms and signs of ischemic heart disease, preserved LVEF and no obstructive CAD undergoing clinically indicated invasive coronary flow reserve (CFR) as a measure of CMD-related ischemia and resting left ventricular end-diastolic pressure (LVEDP). Chi-square testing was used for categorical variables and ANOVA or Kruskal-Wallis tests were used for continuous variables.

**Results:**

Overall, 79% had an elevated resting LVEDP, and mean NT-proBNP was 115 ± 158 pg/mL. NT-proBNP levels correlated directly with age (r = 0.28, p = <0.0001), and indirectly with body mass index (r = -0.21, p = 0.0006), but did not independently associate with CFR. When stratified by NT-proBNP thresholds, higher NT-proBNP was initially associated with lower CFR, which did not persist with adjustment for multiple testing (p = 0.01 and 0.36, respectively).

**Conclusion:**

Among women with symptoms and signs of ischemia, preserved LVEF, no obstructive CAD, and undergoing clinically indicated functional coronary angiography (FCA) for suspected CMD, while a majority had elevated resting LVEDP, we failed to find an independent association between CFR and NT-proBNP, although stratified clinical thresholds may relate to lower CFR. Further work is needed to investigate if these findings support the hypothesis that CMD-related ischemia may be a precursor to HFpEF.

## Introduction

Women with symptoms and signs of myocardial ischemia, preserved left ventricular ejection fraction (LVEF), and no obstructive coronary artery disease (CAD), often have coronary microvascular dysfunction (CMD) [1]. While the mechanistic pathways of CMD are not fully understood, these women are at increased risk of major cardiovascular events, the most prevalent of which is hospitalization for heart failure with preserved ejection fraction (HFpEF) at longer term follow-up [2–4]. This finding has led to the hypothesis that ischemia due to CMD is precursor to the development of HFpEF [5,6].

To address this hypothesis, we investigated the relationship between CMD-related ischemia measured by coronary flow reserve (CFR) and N-terminal pro-B-type natriuretic peptide (NT-proBNP), an established biomarker of clinical heart failure [7–11], which is synthesized and secreted by ventricular cardiomyocytes under periods of increased myocardial wall stress and is also expressed in response to ischemia [12,13]. Further, NT-proBNP levels across a wide range of values non-diagnostic for heart failure (HF) predict mortality in patients with clinically stable CAD [14]. Because women with signs and symptoms of ischemic heart disease (IHD) without obstructive CAD often have elevated resting left ventricular end-diastolic pressures (LVEDP) [15,16] and are at risk for heart failure hospitalizations [2], we hypothesized that NT-proBNP is related to CMD-related ischemia. As part of the Women’s Ischemia Syndrome Evaluation—Coronary Vascular Dysfunction (WISE-CVD) Study, women were evaluated for CMD by clinically indicated invasive functional coronary angiography (FCA). We examined relations between NT-proBNP and CMD in the WISE-CVD women who underwent FCA.

## Methods

We analyzed women with FCA in the National Heart, Lung and Blood Institute (NHLBI)-sponsored WISE-CVD Project (enrolled 2008–2015), a prospective cohort study aimed at improving pathophysiological understanding in women with IHD and angiographically no obstructive CAD. All studies were performed at Cedars-Sinai Medical Center, Los Angeles, or the University of Florida, Gainesville, where institutional review boards approved the project and all participants provided written informed consent.

Inclusion criteria for WISE-CVD included women ≥18 years old, with symptoms (chest pain, shortness of breath) and signs (abnormal stress testing) leading to a clinically determined coronary angiogram by a treating physician, preserved ejection fraction (LVEF >50%), and no history of heart failure, and no obstructive CAD, defined as ≥50% luminal diameter stenosis in ≥1 epicardial artery. Women in the current analysis also underwent clinically indicated FCA (n = 208). Exclusion criteria included acute coronary syndrome, prior percutaneous coronary intervention or bypass surgery, primary valvular heart disease, concurrent cardiogenic shock, chest pain with known non-ischemic pathogenesis, severe renal impairment, and cardiomyopathy (including hypertrophic cardiomyopathy). Participants underwent a baseline physical exam that included heart rate, blood pressure, height, weight, body mass index (BMI) determination, demographics and medical history.

### Functional coronary angiography and invasive hemodynamic measurements

Invasive FCA, with vasoactive agents considered a reference standard for the diagnosis of CMD [17], was performed using a standardized protocol, including resting LVEDP prior to any provocative testing [17]. Briefly, a Doppler wire (Flowire, Volcano Inc) was placed in the proximal left anterior descending artery (LAD). Coronary micro- and macrovascular dilation and constriction pathways were then investigated by infusing intra-coronary adenosine, acetylcholine, and nitroglycerin directly into most often the LAD, as previously described [17,18]. Hemodynamic data, Doppler coronary blood flow (CBF) velocities, and coronary cine angiography were obtained after each infusion. Data were analyzed by the WISE core laboratory, masked to all clinical data. Coronary microvascular dilation dysfunction was defined as an adenosine-induced coronary flow reserve (CFR) <2.5, which we used as a measure of CMD-related ischemia, and has previously been show to predict adverse outcomes in a WISE [19] and other cohort studies [20]. We also evaluated coronary microvascular constriction dysfunction, defined as CBF change < 50% in response to acetylcholine [18], coronary macrovascular constriction dysfunction, defined as a lack of LAD diameter increase to intracoronary acetylcholine (ΔAch) [21], and coronary macrovascular dilation dysfunction, defined as a change in LAD diameter ≤ 20% in response to intracoronary nitroglycerin (ΔNTG). Clinically abnormal resting LVEDP was < 12 mmHg as previously defined [22–24].

### Cardiac magnetic resonance imaging protocol

Cardiac magnetic resonance imaging (CMRI) was performed on all subjects in a supine position on a 1.5-Tesla CMRI scanner (Avanto: Siemens Healthcare, Erlangen, Germany) with ECG gating and a phase-array surface coil (CP Body Array Flex: Seimens Healthcare). A highly standardized protocol was used and included assessment of left ventricular morphology and function. Prior to the CMRI, an intravenous line placement was placed for the infusion of adenosine or regadenoson. Adenosine was used (set at 140mcg/kg/min) unless there was a contraindication to adenosine, in which case regadenoson was used (0.4 mg/5 mL). Pharmacological stress and rest first-pass myocardial perfusion imaging was obtained using gadolinium contrast agent set at 0.05mM/kg. Blood pressure and pulse oxygenation were monitored and recorded before, during and after infusion. A 12 lead ECG was recorded prior to and following the CMRI. CMRI images were downloaded to DIACOM formatted CDs and transmitted to CMRI core laboratory at Cedars-Sinai Medical Center.

### NT-ProBNP

Venous blood samples were collected at rest during the baseline visit. Samples were frozen at -70⁰C and shipped for batch analyses at ICL laboratory (Mayo Clinic, Rochester, MN). NT-proBNP was measured by an automated double incubation sandwich assay on the Roche Cobas e411 (Roche Diagnostics, Indianapolis, IN 46250). Intra-assay coefficient of variation, at increasing NT-proBNP concentrations, are 2.2%, 1.1% and 0.8% at 56.9, 438 and 2092 pg/mL respectively. Inter-assay coefficient of variation is 7.8%, 3.0% and 2.5% at 36.1, 867 and 1986 pg/mL respectively. Clinical reference ranges vary for men versus women and for age; for our cohort of women with a mean age of 53 years, the clinical reference range was 5–155 pg/mL.

### Statistical analysis

Baseline characteristics are summarized as mean ± standard deviation (SD). Pearson correlation using log NT-proBNP was performed for statistical analysis. NT-proBNP was log-transformed due to the skewed distribution in this cohort. Correlation was considered statistically significant if p was < 0.05. Additional NT-proBNP analysis were performed using clinically defined, thresholds, defined in prior studies to correlate with cardiovascular outcomes, according to NT-proBNP levels <100 pg/mL, 100–399 pg/mL, and ≥400 pg/mL [10,11,25–27]. Subject characteristics and measurements of CMD were analyzed in these subgroups. Fisher’s Exact test was used for categorical variables and Kruskal-Wallis tests were used for continuous variables. A Holm-Bonferroni multiple testing correction (ref) was used to adjust all the p-values from the tests among these three pre-defined BNP subgroups in Tables 1 and 2 [28].

**Table 1:** Baseline demographics.

Median and range, Mean±SD, or N (%)	n = 208	NT-pro BNP <100pg/mL n = 144	NT-pro BNP 100-399pg/mL n = 53	NT-pro BNP ≥400pg/mL n = 11	p-value	Holm adjusted p-value
Serum NT-proBNP pg/mL (median, range)	65 (9, 1406)	45 (9,98)	153 (100, 373)	510 (418, 1406)	-	-
Age (years)	53± 11	51 ± 11	58 ±10	61 ±13	**0.0001**	**0.0043**
BMI (kg/m^2^)	31 ± 8	31.5 ± 8	29 ± 8	29 ± 6	0.07	1
Caucasian	145 (70%)	97 (67%)	39 (74%)	9 (83%)	0.54	1
Post-menopausal	135 (65%)	86 (60%)	39 (74%)	10 (91%)	**0.04**	**1**
History of Diabetes	25 (12%)	20 (14%)	4 (8%)	1 (9%)	0.52	1
History of HTN	79 (38%)	48 (33%)	25 (47%)	6 (55%)	0.10	1
History of DYS	25 (12%)	17 (12%)	6 (11%)	2 (18%)	0.75	1
History of Tobacco use (current and former)	92 (44%)	63 (44%)	22 (42%)	7 (64%)	0.40	1
Aspirin	131 (63%)	94 (65%)	28 (53%)	9 (82%)	0.13	1
Statin	70 (34%)	46 (32%)	20 (38%)	4 (36%)	0.73	1
ACE-I or ARB	41 (20%)	27 (19%)	10 (19%)	4 (36%)	0.37	1
Beta Blocker	49 (24%)	28 (19%)	18 (34%)	3 (27%)	0.09	1
Diuretics	31 (15%)	18 (13%)	9 (17%)	4 (36%)	0.08	1
Hormone Therapy	89 (43%)	52 (36%)	30 (57%)	7 (64%)	**0.01**	**0.36**
Systolic Blood Pressure (mmHg)	128 ± 19	127 ± 18	128 ± 20	137 ± 20	0.20	1
Diastolic Blood Pressure (mmHg)	71 ± 11	72 ± 11	68 ± 12	75 ± 13	0.06	1
Total Cholesterol (mg/dl)	184 ± 41	184 ± 42	187 ± 35	165 ± 45	0.53	1
Low Density Lipoprotein (mg/dl)	101 ± 33	103 ± 35	95 ± 28	97 ± 36	0.66	1
High Density Lipoprotein (mg/dl)	58 ± 16	56 ± 15	64 ± 20	60 ± 7	0.13	1
Triglycerides (mg/dl)	143 ± 134	146 ± 140	140 ± 129	99 ± 28	0.85	1
Fasting Glucose (mg/dl)	98 ± 30	100 ± 34	93 ± 16	93 ± 13	0.69	1
Creatinine (mg/dl)	0.85 ± 1.13	0.86 ± 1.32	0.84 ± 0.18	0.77 ± 0.12	**0.01**	**0.36**
Hemoglobin (g/dL)	1 ± 2.1	13.2 ± 2.4	13 ± 1.1	13 ± 1.3	0.73	1

ACE-I = Angiotensin converting enzyme inhibitor, ARB = Angiotensin receptor blocker, BMI = body mass index, HTN = hypertension, DYS = dyslipidemia, NT-pro BNP = N-terminal pro-B-type natriuretic peptide. The tests for categorical variable comparisons across BNP categories was Fisher’s Exact test, the test for continuous variables was Kruskal-Wallis test (non-normal distribution or outliers).

**Table 2:** CMRI and CMD variables by NT-proBNP groups (n = 208).

Mean ±SD or %		NT-pro BNP <100 pg/mL n = 144	NT-pro BNP 100–399 pg/mL n = 53	NT-pro BNP ≥400 pg/mL n = 11	p-value	Holm adjusted p-value
**FCA variables**						
CFR	2.7 ± 0.6	2.8 ± 0.6	2.6 ± 0.6	2.3 ± 0.7	**0.01**	**0.36**
CBF response (%)	67.72 ± 90.35	73.52 ± 95.39	55.43 ± 71.12	31.91 ± 48.58	0.25	1
ΔAch (%)	-9.43 ± 20.07	-10.75 ± 20.72	-6.46 ± 17.84	-6.15 ± 22.4	0.43	1
ΔNTG (%)	5.08 ± 20.84	4.6 ± 20.87	6.75 ± 20.66	2.85 ± 23.61	0.80	1
LVEDP (mmHg)	15 ± 5	15 ± 5	15 ± 5	16 ± 9	0.98	1
**CMRI variables**						
Ejection Fraction %	67±7	67 ± 7	69 ± 8	65 ± 11	0.82	1
Left Ventricular Mass (g)	95±18	95 ±18	89 ± 12	107 ± 30	0.07	1
End Diastolic Volume	124±25	124±26	121±19	126±32	0.80	1
End Systolic Volume	41±14	41±14	38±11	43±17	0.79	1
Stroke Volume	83±18	83±17	83±15	83±30	0.86	1

Ach = acetylcholine, CBF = coronary blood flow, CFR = coronary flow reserve, FCA = functional coronary angiography, LVEDP = left ventricular end diastolic filling pressure, NTG = nitroglycerin, NT-pro BNP = N-terminal pro-B-type natriuretic peptide.

The tests for categorical variable comparisons across BNP categories was Fisher’s Exact test, the test for continuous variables Kruskal-Wallis test (non-normal distribution or outliers).

Multiple linear regression was used to assess the association of the outcome of log transformed NT-proBNP with CFR, adjusting for covariates listed subsequently. Regression analyses were conducted in stages as follows: Model I is unadjusted, Model II is adjusted for age, and body mass index, Model III is adjusted for age, body mass index, education level, fasting glucose, history of hypertension, history of diabetes, history of dyslipidemia, high-density lipoprotein, low-density lipoprotein, creatinine, systolic and diastolic blood pressure, heart rate, medications (antihypertensive, statin and diabetic medications), physical activity and smoking status. All analyses were done using SAS version 9.3 (SAS Institute, Cary, NC).

## Results

Pertinent baseline characteristics are summarized for the total group and stratified by NT-proBNP levels (**Table 1**). Overall group median NT-proBNP was 69 pg/mL (range 9–1406 pg/mL) with 5% (n = 11/208) of subjects having an NT-proBNP >400 pg/mL. A subset of 186 women with complete LVEDP measurement demonstrated that 79% (n = 146/186) had an LVEDP (≥12mmHg), and 25% (n = 46/186) had an LVEDP >18mmHg. Log NT-proBNP directly correlated with age (r = 0.28, p = <0.0001), and inversely with BMI (r = -0.21, p = 0.0006), but not LVEDP.

**Fig 1:**
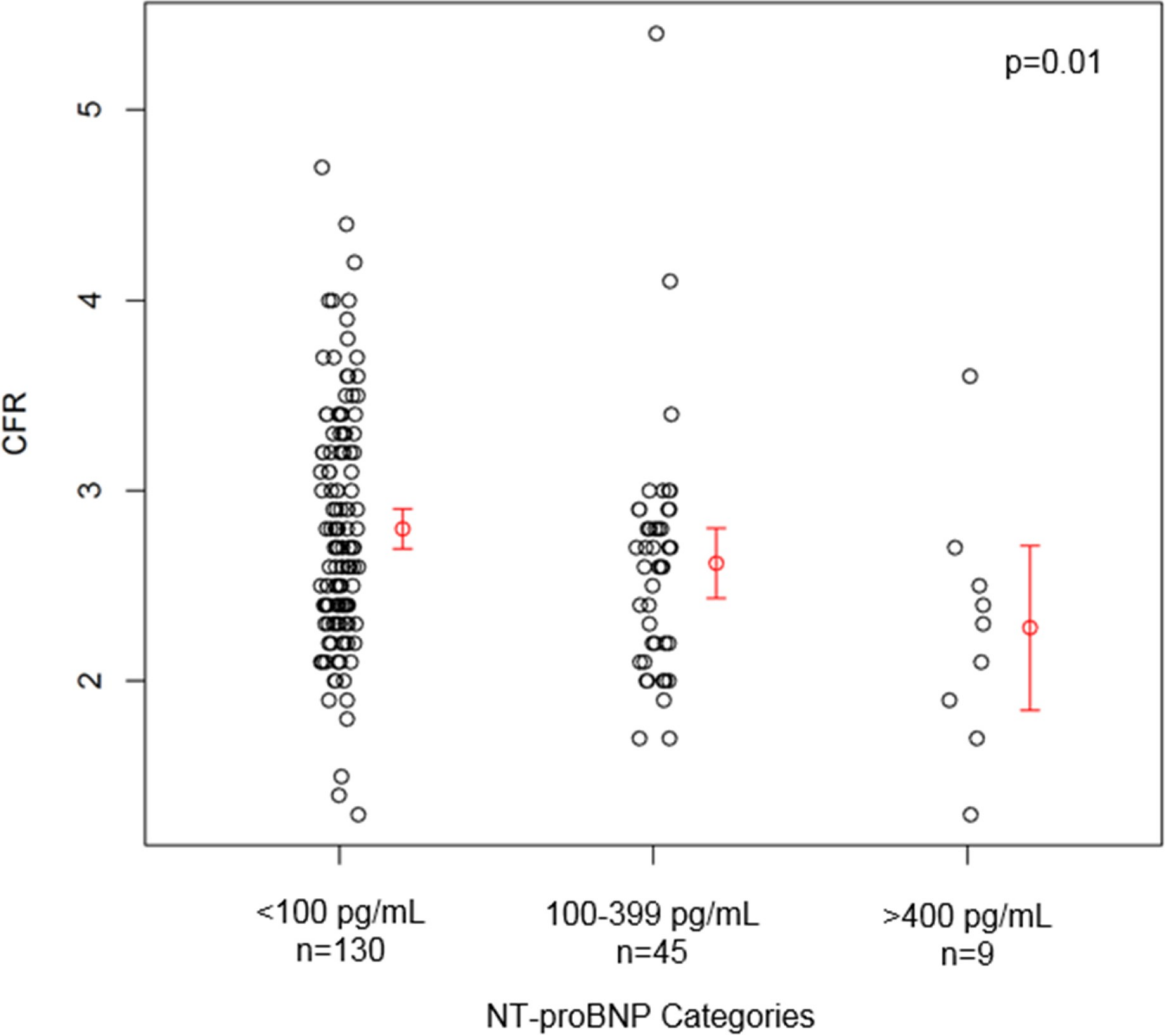
Scatter plot of CFR by NT-proBNP categories. Mean and 95% confidence interval for the mean of each category in red (Kruskal-Wallis test, p = 0.01) (total n = 184).

Analysis using the pre-specified subgroup analysis of clinically-defined thresholds of NT-proBNP (<100, 100–399, and ≥400 pg/mL) [10,11,25–27] revealed graded direct relationships between higher NT-proBNP with age, post-menopausal status, hormone replacement therapy use, diastolic blood pressure and lower creatinine levels (**Table 1**). Further, higher NT-proBNP demonstrated a graded relationship with lower CFR (**Table 2, Fig 1**), although this did not remain statistically significant following multiple test adjustment (p = 0.01, and p = 0.36, respectively, **Table 2**). Notably, this was most evident in the small subgroup of 11 women above the clinically “abnormal” NT-proBNP threshold >400 mg/dL, where 6 (46%) had an abnormal resting LVEDP of >12 mmHg, and 6 (46%) had an abnormal CFR of <2.5.

Linear regression models showed no unadjusted linear association between log NT-proBNP and CFR (estimate -0.17 per unit increase in CFR, p = 0.14). There were consistent associations between log NT-proBNP and age and BMI, respectively, with CFR in the model. Increasing one year of age was associated with 0.029 increase in log NT proBNP (p<0.0001), adjusted for BMI and CFR. Further, an increase of one unit of BMI was associated with a 0.021 decrease in log NT-proBNP (p = 0.017), although CFR had a non-significant decreasing association with log NT proBNP (p = 0.12). Further adjustment by other factors did not show associations between CFR and log NT-proBNP.

## Discussion

Among women with symptoms and signs of ischemia, preserved LVEF, no obstructive CAD, while a majority had elevated resting LVEDP, the current analyses do not support a strong association between CFR and NT-proBNP across the cohort. Although CFR indicative of CMD-related ischemia was lower in the small subgroup of women with elevated NTproBNP >400 mg/dL consistent with heart failure, this was not a common finding. Indeed, given the cohort exclusion criteria of preserved LVEF and no prior history of heart failure, this low prevalence is not surprising. Viewing the full cohort findings, our data suggest that further work is needed to test the hypothesis that CMD-related ischemia may be a precursor to HFpEF in this population.

Our analyses stratified by clinically relevant NT-proBNP levels demonstrating higher NT-proBNP associated with lower CFR are consistent prior reports. Specifically, in the Multi-Ethnic Study of Atherosclerosis (MESA) in a population with a wider range of flow reserve values, myocardial perfusion reserve in asymptomatic subjects free of overt CVD was inversely related to NT-proBNP, suggesting that subclinical CMD is related to elevated levels of NT-proBNP [29]. Notably, this MESA study demonstrated that one of the strongest predictors of NT-proBNP was older age, male sex, and diabetes. Conversely, the WISE-CVD population was middle-aged, all-female, with lower prevalence of diabetes and used CFR to evaluate CMD. However, both results suggest potential relations between CMD and NT-proBNP.

A more recent study by Hirakawa et al. [30] of clinically stable HF patients without obstructive CAD examined relationships between endothelial-independent CMD and plasma B-type natriuretic peptide (BNP) levels, as well as whether each measure correlated with myocardial fibrosis. Trans-cardiac BNP release was determined by sampling from the coronary sinus and aorta while cardiac magnetic resonance imaging evaluated the presence of myocardial scar (late gadolinium enhancement). These investigators also found CFR inversely correlated with plasma BNP levels and trans-cardiac BNP levels, however only among those with late gadolinium enhancement myocardial scar. A BNP level >180 pg/mL independently predicted CFR <2.5 in this population with confirmed heart failure [30]. This group previously reported endothelium-dependent microvascular dysfunction correlated with cardiac fibrosis in heart failure patients without obstructive CAD [31]. Our findings extend these observations, and suggest that ventricular scar and fibrosis may be the relatively greater contributors to future heart failure, compared to ischemia [32].

We found that women with higher NT-proBNP levels and lower CFR were more often post-menopausal and on hormone replacement therapy. There are conflicting data related to menopause and hormone replacement therapy and NT-proBNP. More recent studies have shown that post-menopausal status and estrogen use does not appear to be associated with increased levels of NT-proBNP [33,34]. Other baseline differences in our women with higher NT-proBNP included lower creatinine levels measured at baseline. This is in contrast to previous studies showing higher levels of NT-proBNP related to lower glomerular filtration rate [35,36]. This inconsistency could be due to our unique female-specific population, relatively small sample size, or other unknown factors, and underscore the need for research in women and sex-specific investigation.

In our cohort, while the majority had an elevated resting LVEDP, women with clinical heart failure were excluded, and few women (5%) met the clinical threshold “heart failure” level of NT-proBNP >400pg/mL. The exact mechanism for these elevated LVEDP measurements in these women is not understood. The women were on average overweight, over a third had history of hypertension and 12% diabetes. We have previously observed impaired ventricular diastolic function in women with CMD [37], which we have hypothesized contributes to the elevated LVEDP [37]. Elevated LVEDP is an independent predictor of future HF [38] and correlates with NT-proBNP [39,40]. Interestingly, LVEDP did not relate with NT-proBNP in this analysis, and thus it is possible that NT-proBNP requires a minimum LVEDP elevation or ischemia threshold to be surpassed to become elevated. Previous reports demonstrate poor relations between LVEDP and NT-proBNP in obese patients [41] which was evident in our cohort. This explanation is also consistent with previous reports in asymptomatic patients [39,40].

An NT-proBNP ≥ 400 pg/mL has been associated with higher rates of HF and cardiovascular events in patients with stable obstructive CAD. [11,26] and our results evaluated only the central parts of the curvilinear relationship between CFR and NT-proBNP [42], because healthy women and stage C and D of heart failure were not included in our cohort. However, all levels of NT-proBNP provide prognostic information on all-cause mortality, independent of invasive measures of left ventricular function and severity of obstructive CAD [14]. Our clinically relevant thresholds of NT-proBNP, were associated with reduced CFR, and these thresholds are independent predictors of clinical adverse events [2,43]. Our NT-proBNP positive relations to age and negative relation to BMI are also consistent with previous work [44–47]. Our current results extend these previous observations by demonstrating that relations between age and NT-proBNP appear to be maintained in women younger than previously studied [44,45], and contribute to knowledge gaps regarding CMD, a relatively newer recognized and increasing burden in women and men [48].

### Limitations

Our study is limited by a relatively small sample size, especially those with NT-proBNP level ≥400 pg/dL due to our exclusion of heart failure, in our cohort of women with signs and symptoms of ischemia, preserved LVEF and no obstructive CAD. Because our study is a cross-sectional design of women with a high prevalence of CMD, with a limited range of coronary flow reserves, it is possible that we have underestimated the true extent of the associations. Specifically, we used linear regression analyses to assess the relationship between NT-proBNP and CFR which is curvilinear [42], due to our cohort not including reference control and Class C and D heart failure women. Serial measurements or even trans-myocardial gradients, may provide more detailed information about relations between CMD-related ischemia and NT-proBNP. It is possible that our results were influenced by medications known to lower NT-proBNP, including the use of ACE-inhibitor or angiotensin receptor blocker, beta blocker [49], and hormone replacement therapy that can increase NT-proBNP levels [50]. We purposely did not exclude women on these medications in our analysis because we believe that it is important to test the predictive value of NT-proBNP under “real-life” circumstances; nevertheless, medication use did enter the regression modeling. Further, the aim of the hypothesis testing done was to explore associations of the NT-proBNP in all the women with data available in this cohort. It was not a randomized clinical trial and no control group was available for analysis. We have added a Holm-Bonferroni multiple testing correction for all the hypothesis tests looking at differences among the categorical NT-proBNP subgroups for potential confounding. The only significant difference among these groups after applying this correction was in age. Finally, LVEDP and NT-proBNP were measured at fasting, resting conditions, while stress-LVEDP would be more sensitive and diagnostic for heart failure [51].

## Conclusions

Among women with symptoms and signs of ischemia, preserved LVEF and no obstructive CAD, and undergoing clinically-indicated FCA for suspected CMD, while a majority had elevated resting LVEDP, we failed to find an association between CFR and NT-proBNP. CFR indicative of CMD-related ischemia was lower when stratified by clinical thresholds of NT-proBNP in the small subgroup of women with elevated NTproBNP >400 mg/dL consistent with heart failure, however this was not a common finding. Further work is needed to investigate if these findings support the hypothesis that CMD-related ischemia may be a precursor to HFpEF in this population and require further investigation.

## Supporting information

S1 File (CSV)

## References

[1] Reis SE, Holubkov R, Smith A, Kelsey SF, Sharaf BL, Reichek N, et al Coronary microvascular dysfunction is highly prevalent in women with chest pain in the absence of coronary artery disease: results from the NHLBI WISE study. American heart journal. 2001;141(5):735–41. 10.1067/mhj.2001.114198 11320360

[2] Gulati M, Cooper-DeHoff RM, McClure C, Johnson BD, Shaw LJ, Handberg EM, et al Adverse cardiovascular outcomes in women with nonobstructive coronary artery disease: a report from the Women's Ischemia Syndrome Evaluation Study and the St James Women Take Heart Project. Archives of internal medicine. 2009;169(9):843–50. 10.1001/archinternmed.2009.50 19433695 PMC2782882

[3] Taqueti VR, Di Carli MF. Response by Taqueti and Di Carli to Letter Regarding Article, "Excess Cardiovascular Risk in Women Relative to Men Referred for Coronary Angiography Is Associated With Severely Impaired Coronary Flow Reserve, Not Obstructive Disease". Circulation. 2017;136(2):241–2. 10.1161/CIRCULATIONAHA.117.028569 28696272

[4] Bakir M, Nelson MD, Jones E, Li Q, Wei J, Sharif B, et al Heart failure hospitalization in women with signs and symptoms of ischemia: A report from the women's ischemia syndrome evaluation study. Int J Cardiol. 2016;223:936–9. 10.1016/j.ijcard.2016.07.301 27589041 PMC8312227

[5] Pepine CJ, Petersen JW, Merz CNB. A Microvascular-Myocardial Diastolic Dysfunctional State and Risk for Mental Stress Ischemia: A Revised Concept of Ischemia During Daily Life*. JACC: Cardiovascular Imaging. 2014;7(4):362–5. 10.1016/j.jcmg.2013.11.009 24742891

[6] Paulus WJ, Tschöpe C. A novel paradigm for heart failure with preserved ejection fraction: comorbidities drive myocardial dysfunction and remodeling through coronary microvascular endothelial inflammation. Journal of the American College of Cardiology. 2013;62(4):263–71. 10.1016/j.jacc.2013.02.092 23684677

[7] Booth RA, Hill SA, Don-Wauchope A, Santaguida PL, Oremus M, McKelvie R, et al Performance of BNP and NT-proBNP for diagnosis of heart failure in primary care patients: a systematic review. Heart failure reviews. 2014;19(4):439–51. 10.1007/s10741-014-9445-8 24969534

[8] Weber M, Hamm C. Role of B-type natriuretic peptide (BNP) and NT-proBNP in clinical routine. Heart. 2006;92(6):843–9. 10.1136/hrt.2005.071233 16698841 PMC1860679

[9] Oremus M, Don-Wauchope A, McKelvie R, Santaguida PL, Hill S, Balion C, et al BNP and NT-proBNP as prognostic markers in persons with chronic stable heart failure. Heart failure reviews. 2014;19(4):471–505. 10.1007/s10741-014-9439-6 24986335

[10] McKie PM, Rodeheffer RJ, Cataliotti A, Martin FL, Urban LH, Mahoney DW, et al Amino-Terminal Pro-B-Type Natriuretic Peptide and B-Type Natriuretic Peptide Biomarkers for Mortality in a Large Community-Based Cohort Free of Heart Failure. Hypertension. 2006;47(5):874–80. 10.1161/01.HYP.0000216794.24161.8c 16585413 PMC2647805

[11] Mishra RK, Beatty AL, Jaganath R, Regan M, Wu AH, Whooley MA. B‐type Natriuretic Peptides for the Prediction of Cardiovascular Events in Patients With Stable Coronary Heart Disease: The Heart and Soul Study. Journal of the American Heart Association. 2014;3(4):e000907 10.1161/JAHA.114.000907 25053234 PMC4310375

[12] Goetze JP, Christoffersen C, Perko M, Arendrup H, Rehfeld JF, Kastrup J, et al Increased cardiac BNP expression associated with myocardial ischemia. FASEB J. 2003;17(9):1105–7. 10.1096/fj.02-0796fje 12709407

[13] D'Souza SP, Davis M, Baxter GF. Autocrine and paracrine actions of natriuretic peptides in the heart. Pharmacol Ther. 2004;101(2):113–29. 10.1016/j.pharmthera.2003.11.001 14761702

[14] Kragelund C, Grønning B, Køber L, Hildebrandt P, Steffensen R. N-terminal pro–B-type natriuretic peptide and long-term mortality in stable coronary heart disease. New England Journal of Medicine. 2005;352(7):666–75. 10.1056/NEJMoa042330 15716560

[15] Goykhman P, Mehta P, Kothawade K, Yang YC, Kar S, Samuels BA, et al Elevated Left Ventricular End Diastolic Pressure and Microvascular Coronary Dysfunction. Circulation. 2010;122(21 Supplement):A15881.

[16] Elhabyan A-K, Reyes BJ, Hallak O, Broce M, Rosencrance JG, Lucas BD, et al Subendocardial ischemia without coronary artery disease: is elevated left ventricular end diastolic pressure the culprit? Current Medical Research and Opinion®. 2004;20(5):773–7. 10.1185/030079904125003359 15140345

[17] Wei J, Mehta PK, Johnson BD, Samuels B, Kar S, Anderson RD, et al Safety of coronary reactivity testing in women with no obstructive coronary artery disease: results from the NHLBI-sponsored WISE (women's ischemia syndrome evaluation) study. JACC: Cardiovascular Interventions. 2012;5(6):646–53. 10.1016/j.jcin.2012.01.023 22721660 PMC3417766

[18] von Mering GO, Arant CB, Wessel TR, McGorray SP, Merz CNB, Sharaf BL, et al Abnormal Coronary Vasomotion as a Prognostic Indicator of Cardiovascular Events in Women Results From the National Heart, Lung, and Blood Institute–Sponsored Women’s Ischemia Syndrome Evaluation (WISE). Circulation. 2004;109(6):722–5. 10.1161/01.CIR.0000115525.92645.16 14970106

[19] Pepine CJ, Anderson RD, Sharaf BL, Reis SE, Smith KM, Handberg EM, et al Coronary Microvascular Reactivity to Adenosine Predicts Adverse Outcome in Women Evaluated for Suspected IschemiaResults From the National Heart, Lung and Blood Institute WISE (Women's Ischemia Syndrome Evaluation) Study. Journal of the American College of Cardiology. 2010;55(25):2825–32. 10.1016/j.jacc.2010.01.054 20579539 PMC2898523

[20] Britten MB, Zeiher AM, Schächinger V. Microvascular dysfunction in angiographically normal or mildly diseased coronary arteries predicts adverse cardiovascular long-term outcome. Coron Artery Dis. 2004;15(5):259–64. 10.1097/01.mca.0000134590.99841.81 15238822

[21] Halcox JP, Schenke WH, Zalos G, Mincemoyer R, Prasad A, Waclawiw MA, et al Prognostic value of coronary vascular endothelial dysfunction. Circulation. 2002;106(6):653–8. 10.1161/01.cir.0000025404.78001.d8 12163423

[22] Pepine C, Hill J, Lambert C. History of the development and application of cardiac catheterization. Diagnostic and Therapeutic Cardiac Catheterization Baltimore: Williams & Wilkins. 1989:3–9.

[23] Davidson CJ, Bonow RO. Cardiac catheterization. Heart disease 5th ed Philadelphia: WB Saunders 1997:177–203.

[24] Mann DL, Zipes DP, Libby P, Bonow RO. Braunwald's heart disease: a textbook of cardiovascular medicine: Elsevier Health Sciences; 2014.

[25] Bibbins-Domingo K, Gupta R, Na B, Wu AH, Schiller NB, Whooley MA. N-terminal fragment of the prohormone brain-type natriuretic peptide (NT-proBNP), cardiovascular events, and mortality in patients with stable coronary heart disease. JAMA. 2007;297(2):169–76. 10.1001/jama.297.2.169 17213400 PMC2848442

[26] Omland T, Pfeffer MA, Solomon SD, de Lemos JA, Røsjø H, Šaltytė Benth J, et al Prognostic value of cardiac troponin I measured with a highly sensitive assay in patients with stable coronary artery disease. J Am Coll Cardiol. 2013;61(12):1240–9. 10.1016/j.jacc.2012.12.026 23414791

[27] Wang TJ, Larson MG, Levy D, Benjamin EJ, Leip EP, Omland T, et al Plasma natriuretic peptide levels and the risk of cardiovascular events and death. N Engl J Med. 2004;350(7):655–63. 10.1056/NEJMoa031994 14960742

[28] Holm S. A simple sequentially rejective multiple test procedure. Scandinavian Journal of Statistics. 1979;6:65–70.

[29] Mitchell A, Misialek JR, Folsom AR, Duprez D, Alonso A, Jerosch-Herold M, et al Usefulness of N-terminal Pro-brain Natriuretic Peptide and Myocardial Perfusion in Asymptomatic Adults (from the Multi-Ethnic Study of Atherosclerosis). The American journal of cardiology. 2015;115(10):1341–5. 10.1016/j.amjcard.2015.02.040 25816778 PMC4414796

[30] Hirakawa K, Yamamuro M, Uemura T, Takashio S, Kaikita K, Utsunomiya D, et al Correlation between microvascular dysfunction and B-type natriuretic peptide levels in non-ischemic heart failure patients with cardiac fibrosis. Int J Cardiol. 2017;228:881–5. 10.1016/j.ijcard.2016.11.054 27889555

[31] Uemura T, Yamamuro M, Kaikita K, Takashio S, Utsunomiya D, Hirakawa K, et al Late gadolinium enhancement on cardiac magnetic resonance predicts coronary vasomotor abnormality and myocardial lactate production in patients with chronic heart failure. Heart Vessels. 2016;31(12):1969–79. 10.1007/s00380-016-0816-z 26892530 PMC5122619

[32] Nelson MD, Wei J, Bairey Merz CN. Coronary microvascular dysfunction and heart failure with preserved ejection fraction as female-pattern cardiovascular disease: the chicken or the egg? Eur Heart J. 2018;39(10):850–2. 10.1093/eurheartj/ehx818 29346550 PMC5939623

[33] Chang AY, Abdullah SM, Jain T, Stanek HG, Das SR, McGuire DK, et al Associations among androgens, estrogens, and natriuretic peptides in young women: observations from the Dallas Heart Study. J Am Coll Cardiol. 2007;49(1):109–16. 10.1016/j.jacc.2006.10.040 17207730

[34] Lam CS, Cheng S, Choong K, Larson MG, Murabito JM, Newton-Cheh C, et al Influence of sex and hormone status on circulating natriuretic peptides. J Am Coll Cardiol. 2011;58(6):618–26. 10.1016/j.jacc.2011.03.042 21798425 PMC3170816

[35] Horii M, Matsumoto T, Uemura S, Sugawara Y, Takitsume A, Ueda T, et al Prognostic value of B-type natriuretic peptide and its amino-terminal proBNP fragment for cardiovascular events with stratification by renal function. J Cardiol. 2013;61(6):410–6. 10.1016/j.jjcc.2013.01.015 23618914

[36] Takase H, Dohi Y. Kidney function crucially affects B-type natriuretic peptide (BNP), N-terminal proBNP and their relationship. Eur J Clin Invest. 2014;44(3):303–8. 10.1111/eci.12234 24372567

[37] Nelson MD, Szczepaniak LS, Wei J, Haftabaradaren A, Bharadwaj M, Sharif B, et al Diastolic Dysfunction in Women With Signs and Symptoms of Ischemia in the Absence of Obstructive Coronary Artery Disease: A Hypothesis-Generating Study. Circulation: Cardiovascular Imaging. 2014;7(3):510–6. 10.1161/CIRCIMAGING.114.001714 24633782 PMC4031259

[38] Liang H-Y, Cauduro SA, Pellikka PA, Bailey KR, Grossardt BR, Yang EH, et al Comparison of usefulness of echocardiographic Doppler variables to left ventricular end-diastolic pressure in predicting future heart failure events. The American journal of cardiology. 2006;97(6):866–71. 10.1016/j.amjcard.2005.09.136 16516591

[39] Tschöpe C, Kašner M, Westermann D, Gaub R, Poller WC, Schultheiss H-P. The role of NT-proBNP in the diagnostics of isolated diastolic dysfunction: correlation with echocardiographic and invasive measurements. European heart journal. 2005;26(21):2277–84. 10.1093/eurheartj/ehi406 16014646

[40] Gremmler B, Kunert M, Schleiting H, Kisters K, Ulbricht LJ. Relation between N-terminal pro-brain natriuretic peptide values and invasively measured left ventricular hemodynamic indices. Experimental & Clinical Cardiology. 2003;8(2):91 19641656 PMC2716205

[41] Taylor JA, Christenson RH, Rao K, Jorge M, Gottlieb SS. B-type natriuretic peptide and N-terminal pro B-type natriuretic peptide are depressed in obesity despite higher left ventricular end diastolic pressures. Am Heart J. 2006;152(6):1071–6. 10.1016/j.ahj.2006.07.010 17161055

[42] Emdin M, Passino C, Prontera C, Fontana M, Poletti R, Gabutti A, et al Comparison of brain natriuretic peptide (BNP) and amino-terminal ProBNP for early diagnosis of heart failure. Clin Chem. 2007;53(7):1289–97. 10.1373/clinchem.2006.080234 17495021

[43] Taqueti VR, Hachamovitch R, Murthy VL, Naya M, Foster CR, Hainer J, et al Global Coronary Flow Reserve Is Associated With Adverse Cardiovascular Events Independently of Luminal Angiographic Severity and Modifies the Effect of Early Revascularization. Circulation. 2015;131(1):19–27. 10.1161/CIRCULATIONAHA.114.011939 25400060 PMC4286486

[44] Sanchez OA, Duprez DA, Bahrami H, Daniels LB, Folsom AR, Lima JA, et al The associations between metabolic variables and NT-proBNP are blunted at pathological ranges: The Multi-Ethnic Study of Atherosclerosis. Metabolism. 2014;63(4):475–83. 10.1016/j.metabol.2013.11.017 24388001 PMC3965618

[45] März W, Tiran B, Seelhorst U, Wellnitz B, Bauersachs J, Winkelmann BR, et al N-terminal pro-B-type natriuretic peptide predicts total and cardiovascular mortality in individuals with or without stable coronary artery disease: the Ludwigshafen Risk and Cardiovascular Health Study. Clinical chemistry. 2007;53(6):1075–83. 10.1373/clinchem.2006.075929 17446333

[46] Wang TJ, Larson MG, Levy D, Benjamin EJ, Leip EP, Wilson PW, et al Impact of obesity on plasma natriuretic peptide levels. Circulation. 2004;109(5):594–600. 10.1161/01.CIR.0000112582.16683.EA 14769680

[47] Fradley MG, Larson MG, Cheng S, McCabe E, Coglianese E, Shah RV, et al Reference limits for N-terminal-pro-B-type natriuretic peptide in healthy individuals (from the Framingham Heart Study). The American journal of cardiology. 2011;108(9):1341–5. 10.1016/j.amjcard.2011.06.057 21864812 PMC3209520

[48] Bairey Merz CN, Pepine CJ, Walsh MN, Fleg JL. Ischemia and No Obstructive Coronary Artery Disease (INOCA): Developing Evidence-Based Therapies and Research Agenda for the Next Decade. Circulation. 2017;135(11):1075–92. 10.1161/CIRCULATIONAHA.116.024534 28289007 PMC5385930

[49] Balion CM, Santaguida P, McKelvie R, Hill SA, McQueen MJ, Worster A, et al Physiological, pathological, pharmacological, biochemical and hematological factors affecting BNP and NT-proBNP. Clinical Biochemistry. 2008;41(4):231–9. 10.1016/j.clinbiochem.2007.10.005 17967418

[50] Karjalainen AH, Ruskoaho H, Vuolteenaho O, Heikkinen JE, Bäckström AC, Savolainen MJ, et al Effects of estrogen replacement therapy on natriuretic peptides and blood pressure. Maturitas. 2004;47(3):201–8. 10.1016/S0378-5122(03)00279-2 15036490

[51] Andersen MJ, Borlaug BA. Invasive Hemodynamic Characterization of Heart Failure with Preserved Ejection Fraction. Heart failure clinics. 2014;10(3):435–44. 10.1016/j.hfc.2014.03.001 24975907

